# Evaluation of VNTR polymorphisms of dopamine transporter gene and the risk of bipolar disorder in Zahedan, southeast Iran

**DOI:** 10.22099/mbrc.2017.25056.1261

**Published:** 2017-09

**Authors:** Mansour Shakiba, Mohammad Hashemi, Salah Mahdar, Zahra Rahbari, Gholamreza Bahari

**Affiliations:** 1 Health promotion research Center, Zahedan University of Medical Sciences, Zahedan, Iran; 2 Department of Psychiatry, School of Medicine, Zahedan University of Medical Sciences, Zahedan, Iran; 3 Cellular and Molecular Research Center, Zahedan University of Medical Sciences, Zahedan, Iran; 4 Department of Clinical Biochemistry, School of Medicine, Zahedan University of Medical Sciences, Zahedan, Iran

**Keywords:** Bipolar disorder, dopamine transporter, DAT1, VNTR, polymorphism

## Abstract

The exact role of dopamine transporter gene (DAT1) in the pathogenesis of bipolar disorder type 1 (BD) is not understood. In the present study, we aimed to evaluate the possible association between 30, 40 and 63 bp variable number tandem repeat (VNTR) polymorphisms of *DAT1* gene and the risk of type 1 (BD) in a sample of Iranian population. This case-control study was performed on 152 BD patients and 153 psychiatrically healthy subjects. Genotyping of the variant was done by polymerase chain reaction method. Totally, the findings did not support an association between *DAT1* VNTR polymorphisms and the risk of BD in a sample of southeast Iranian population.

## INTRODUCTION

Bipolar disorder (BD), is an impairing mood disorder that affects about 1% of the population which is characterized by recurrent episodes of hypomania or mania and depression [1]. The dopamine transporter (DAT) is a presynaptic plasma membrane protein and responsible for reuptake of the dopamine from the synaptic cleft [2]. It is encoded by the (solute carrier family 6 (neurotransmitter transporter), member 3) gene (OMIM:126455, *SLC6A3*) which mapped to chromosome 5 (5p15.3) and show variable number tandem repeat (VNTR) polymorphisms [3]. It has been shown that polymorphisms of *DAT1* (OMIM:126455) may play a role in predisposition to BD disorder [4-7]. In the present study, we aimed to evaluate the impact of 30, 40 and 63 bp VNTR polymorphisms of* DAT1* on type 1 (BD) in a sample of Iranian population.

## MATERIALS AND METHODS

This case-control study was performed on 152 confirmed patients with bipolar disorder type 1 (BD) and 153 healthy subjects. The patients were selected from BD admitted to a university-affiliated hospital (Baharan Psychiatric Hospital, Zahedan, Iran). BD was diagnosed according to DSM-IV-TR (Diagnostic and Statistical Manual of Mental Disorders, Fourth Edition, Text Revision) criteria. 

The controls were unrelated healthy subjects with no clinical symptoms or family histories of BD belonged to same ethnicity as patients and living in the same area as the patients (Southeast Iran). The project was approved by Ethics Committee of the Zahedan University of Medical Sciences and informed consent was taken from all subjects. 

DNA was extracted from whole blood samples using salting out method. Genotyping of the 30 bp (rs3836790), 40 bp (rs28363170) and 63 bp VNTR polymorphisms of DAT1 was done by polymerase chain reaction (PCR) method [8-10]. 

Statistical analysis was done by statistical package SPSS 22 software. Data were analyzed by independent sample t-test or χ2 test according to the data. A p-value less than 0.05 were considered statistically significant.

## RESULTS AND DISCUSSION

A total of 305 subjects including 152 confirmed patients with BD1 (110 males, 42 females, mean age 35.0±10.9) and 153 unrelated healthy subjects (108 males, 45 females, mean age 34.1±11.1) were enrolled in the study. The cases and controls were match regarding age and sex (p value=0.463 and 0.731 respectively). 

Table 1 shows the genotype and allele frequencies of *DAT1 *VNTR polymorphisms in type 1 (BD) patients and controls. The findings revealed the 30, 40 as well as 63 bp VNTR polymorphisms of *DAT1* gene were not associated with the risk of BD. 

It has been proposed that dopaminergic dysfunction contribute to the etiology of mood disorders. Dopamine is a vital neurotransmitter essential for normal brain development and function. The DAT1 is important for active reuptake of dopamine released into the synaptic cleft and ends dopamine neurotransmission. Several studies have investigated the impact of *DAT1* gene polymorphisms on BD [4, 5, 7, 11-14], however, the findings were inconsistent.

In summary, the findings of the present study did not support an association between 30, 40 and 63 bp VNTR polymorphisms of *DAT1* gene and the risk of bipolar disorders in a sample of Iranian population. Further studies with larger sample sizes and different ethnicities are required to verify our findings.

**Table 1:** Genotype and allele frequencies of the *DAT1 *VNTR polymorphism in bipolar disorder and healthy subjects

**VNTR polymorphisms**	** Cases n (%)**	**Control n (%)**
**30-bp VNTR**		
5R/5R	92 (60.5)	80 (52.2)
5R/6R	42 (27.6)	63 (41.1)
6R/6R	10 (6.6)	5 (3.3)
6R/11R	1 (0.7)	1 (0.7)
6R/12R	5 (3.3)	3 (2.0)
5R/13R	2 (1.3)	1 (0.7)
χ2=7.53, df=5, p=0.183		
Alleles		
5R	228 (75.0)	224 (73.2)
6R	68 (22.4)	77 (25.2)
11R	1 (0.3)	1 (0.3)
12R	5 (1.6)	3 (1.0)
13R	2 (0.7)	1 (0.3)
χ2=1.42, df=4, p=0.840		
**40-bp VNTR**		
11R/11R	76 (50.0)	82 (53.6)
11R/10R	64 (42.1)	65 (42.4)
10R/10R	9 (5.9)	4 (2.6)
11R/6R	3 (2.0)	1 (0.7)
11R/16R	0 (0.0)	1 (0.7)
χ2=4.15, df=4, p=0.385		
Alleles		
11	219 (72.1)	231 (75.5)
10	82 (27.0)	73 (23.9)
6	3 (0.9)	1 (0.3)
16	0 (0.0)	1 (0.3)
χ2=2.83, df=3, p=0.417		
**63-bp VNTR**		
7R/7R	76 (50.0)	72 (47.1)
7R/8R	59 (38.8)	65 (42.5)
8R/8R	17 (11.2)	16 (10.4)
χ2=2.56, df=2, p=0.633		
Alleles		
7R	211 (69.4)	209 (68.3)
8R	93 (30.6)	97 (34.7)
χ2=0.087, df=1, p=0.295		
